# One-step fabrication of biocompatible chitosan-coated ZnS and ZnS:Mn^2+ ^quantum dots via a γ-radiation route

**DOI:** 10.1186/1556-276X-6-591

**Published:** 2011-11-14

**Authors:** Shu-quan Chang, Bin Kang, Yao-dong Dai, Hong-xu Zhang, Da Chen

**Affiliations:** 1 College of Material Science and Technology, Nanjing University of Aeronautics and Astronautics, Nanjing, 210016, People's Republic of China

**Keywords:** quantum dots, γ-radiation, chitosan, ZnS, ZnS:Mn^2+^, biocompatible

## Abstract

Biocompatible chitosan-coated ZnS quantum dots [CS-ZnS QDs] and chitosan-coated ZnS:Mn^2+ ^quantum dots [CS-ZnS:Mn^2+ ^QDs] were successfully fabricated via a convenient one-step γ-radiation route. The as-obtained QDs were around 5 nm in diameter with excellent water-solubility. These QDs emitting strong visible blue or orange light under UV excitation were successfully used as labels for PANC-1 cells. The cell experiments revealed that CS-ZnS and CS-ZnS:Mn^2+ ^QDs showed low cytotoxicity and good biocompatibility, which offered possibilities for further biomedical applications. Moreover, this convenient synthesis strategy could be extended to fabricate other nanoparticles coated with chitosan.

**PACS: **81.07.Ta; 78.67.Hc; 82.35.Np; 87.85.Rs.

## Introduction

Recently, quantum dots [QDs] have shown great potential in biomedical applications [1-3]. How to minimize the cytotoxicity of QDs is still a great challenge for their clinical applications [4-6]. In the past few years, great efforts have been paid to reduce the toxicity of QDs. Various coatings, such as ZnS shell, thiol-containing molecules, PEG, and other polymers, have been developed to modify the QDs to reduce their cytotoxicity [7-10]. Moreover, a series of non-heavy-metal QDs, such as InGaP/ZnS, ZnS, and ZnS:Mn^2+^, have also been investigated to solve the toxicity problem [11-13]. Significant progresses have been made in producing high quality ZnS and ZnS:Mn^2+ ^QDs by wet chemical methods [14-18]. However, most of these synthesis strategies require the use of an environmentally hazardous organic solvent or surfactant, which is not environmentally friendly and might not be suitable for biomedical applications.

Compared with traditional chemical methods, γ-radiation route, in which reactions can take place in an aqueous system at room temperature under ambient pressure, has been considered as a promising way to process traditional materials or synthesize new materials. Up to now, the γ-radiation route has been employed to prepare many types of novel nanomaterials, such as silver nanoparticles, CdS nanocrystal-modified fibers, CdSe quantum dots, and polyelectrolyte complex [19-23]. Herein, we present a novel strategy to fabricate chitosan-coated ZnS [CS-ZnS] and ZnS:Mn^2+ ^[CS-ZnS:Mn^2+^] quantum dots [QDs] via γ-radiation for the first time.

In our synthesis experiments, chitosan oligomer, a kind of biocompatible polymer, was used as a stabilizer as well as coating molecules [24,25]. By controlling the reaction conditions, the particle sizes and fluorescent properties of QDs could be well tuned. The obtained QDs were around 5 nm in diameter and exhibited excellent fluorescent properties and water solubility. These QDs had low cytotoxicity and high cell membrane penetrability, which offered potentials for biomedical applications.

## Experimental details

### γ-Radiation synthesis of CS-ZnS and CS-ZnS:Mn^2+ ^QDs

CS-ZnS and CS-ZnS:Mn^2+ ^QDs were directly fabricated in an aqueous system at room temperature under ambient pressure via a convenient γ-irradiation route. Chitosan oligomer, with the average polymerization degree of 30, was prepared through radiation degradation [26]. All other reagents used in this study were of analytical grade. In a typical synthesis, 5 mL Zn(Ac)_2 _(10 mM), 5 mL Mn(Ac)_2 _(0.1 mM), and 10 mL Na_2_S_2_O_3 _(10 mM) were added into 70 mL of deionized water containing 0.1% chitosan (*w*/*v*, pH = 5.4) or 2% chitosan (*w*/*v*, pH = 5); then 10 mL (CH_3_)_2_CHOH was added into the above solution as a free radical scavenger. Afterwards, the solution was bubbled with pure nitrogen for 30 min to remove the dissolved oxygen; then the mixed solution was placed in the field of a 1.1 × 10^15 ^Bq ^60^Co γ-ray source with a radiation dose rate of 3 kGy h^-1^. The radiation dose was set as 5, 10, 20, and 30 kGy, respectively. After irradiation, the synthesized QDs were separated from the solution with a centrifugation at 10,000 rpm for 5 min. The samples were washed three times with deionized water and stored in deionized water for further experiments. Thioglycolic acid-coated CdSe QDs [TGA-CdSe QDs] were also synthesized following the reported method [23].

### Characterizations

Transmission electron microscope [TEM] and high-resolution TEM [HRTEM] images were taken using a JEOL JEM-200CX and JEM-2010 (JEOL Ltd., Akishima, Tokyo, Japan). Elementary analysis was carried out with an energy dispersion spectrum [EDS]. X-ray diffraction [XRD] patterns were recorded on a BRUKER D8-ADVANCE (BRUKER AXS GMBH, Karlsruhe, Germany) X-ray diffractometer. Ultraviolet-visible [UV-Vis] spectra were carried out using a PerkinElmer (Waltham, MA, USA) λ-17 spectrophotometer. The photoluminescence [PL] spectra were taken with a Hitachi 850 spectrofluorophotometer (Hitachi High-Tech, Minato-ku, Tokyo, Japan). The quantum yield [QY] values of QDs were calculated relative to rhodamine 6G in water using the following equation: QY_s _= (*F*_s/_*F*_r_) × (*A*_r/_*A*_s_) × QY_r_, where *F *and *A *were the measured fluorescence (area under the emission peak) and the absorbance at the excitation wavelength, respectively. Human pancreatic carcinoma cells (PANC-1, ATCC no. TIB-222) were cultured in a DMEM medium containing 10% (*v*/*v*) fetal calf serum at 37°C in humidified air containing 5% CO_2_. QDs dispersed in phosphate-buffered saline [PBS] were added to the culture medium to achieve a final concentration of 10, 50, 200 or 1,000 μg mL^-1^. An Olympus FV-1000 (Olympus Corporation, Shinjuku-ku, Tokyo, Japan) laser scanning confocal microscope [LSCM] was used to examine the PANC-1 cells labeled with QDs. For LSCM imaging, cells were seeded onto a glass cover slip placed in six well plates. After incubation in a medium containing QDs for 2 h, the PANC-1 cells were fixed with 4% paraformaldehyde for 15 min, and then washed three times with PBS buffer. To clearly observe the distribution and position of QDs in cells, nuclei were stained with 5 μg mL^-1 ^of propidium iodide [PI] for 5 min. Before LSCM imaging, cells were washed with PBS again. MTT assays were performed to assess the metabolic activity of cells labeled with QDs. For MTT assays, cells were cultured in 96 well plates. Before MTT assays, cells were incubated in a medium containing QDs for 2 h, and then the medium was removed and replaced with a serum-free medium (200 μL/well). A total of 20 μL stock MTT (5 mg mL^-1^) was added to each well, and the cells were then incubated for 1 h at 37°C. The medium was removed, and the cells were lysed with DMSO. The absorbance was measured at 595 nm.

## Results and discussion

### Strategy and mechanism for the preparation of CS-ZnS and CS-ZnS:Mn^2+ ^QDs

**Figure 1:**
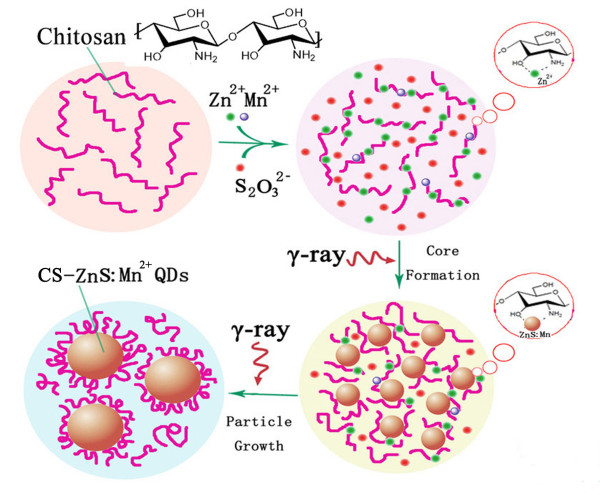
**Scheme of synthesis strategy and mechanism of CS-ZnS and CS-ZnS:Mn^2+ ^QDs via a γ-radiation route**.

Figure 1 illustrated the basic strategy and mechanism for the preparation of CS-ZnS and CS-ZnS:Mn^2+ ^QDs. Chitosan was used as a stabilizer as well as coating molecules in the synthesis processes. (CH_3_)_2_CHOH and excess S_2_O_3_^2- ^were necessary in order to obtain high quality products. First, Zn^2+ ^and Mn^2+ ^were arrested by chitosan through the interaction with -OH and -NH_2 _binding sites and other chemical structures [27]. Subsequently, H_2_O was decomposed by the γ-ray [19]. (CH_3_)_2_CHOH was used to eliminate the strongly oxidative free radical HO· and H_2_O_2_. Hydrated electron and hydrogen atoms with reduction potentials of -2.77 V and -2.13 V were used to reduce S_2_O_3_^2- ^and generate S^2-^. Then, S^2- ^reacted with the bounded Zn^2+ ^and Mn^2+^, and CS-ZnS:Mn^2+ ^QDs were successfully produced. The main reaction processes were proposed as follows:

$${\text{H}}_{\text{2}}\text{O }\overset{\text{γ}\text{ - ray}}{\underset{}{\to }}\;{e_{\text{aq}}}^{-},\;{\text{H}}_{\text{2}}{\text{O}}^{+},\text{ H}∙,\text{ HO}∙,\;{\text{H}}_{\text{2}},\;{\text{H}}_{\text{2}}{\text{O}}_{\text{2}},$$

$${e_{\text{aq}}}^{-}+\;{\text{H}}^{+}\to \text{H}∙,$$

$${\left(\text{C}{\text{H}}_{\text{3}}\right)}_{\text{2}}\text{CHOH}+\text{HO}∙\to \;{\left(\text{C}{\text{H}}_{\text{3}}\right)}_{\text{2}}\left(\text{HO}\right)\text{C}∙+{\text{H}}_{\text{2}}\text{O,}$$

$${\left(\text{C}{\text{H}}_{\text{3}}\right)}_{\text{2}}\left(\text{HO}\right)\text{C}∙\;+\;{\text{H}}_{\text{2}}{\text{O}}_{\text{2}}\to {\left(\text{C}{\text{H}}_{\text{3}}\right)}_{\text{2}}\text{C}=\text{O }+\text{ HO}∙\;+{\text{H}}_{\text{2}}\text{O,}$$

$$\text{2}{\left(\text{C}{\text{H}}_{\text{3}}\right)}_{\text{2}}\left(\text{HO}\right)\text{C}∙\to {\left(\text{C}{\text{H}}_{\text{3}}\right)}_{\text{2}}\text{C}=\text{O }+\;{\left(\text{C}{\text{H}}_{\text{3}}\right)}_{\text{2}}\text{CHOH,}$$

$${\text{S}}_{\text{2}}{\text{O}}_{3}^{2-}+\;\text{2}{e_{\text{aq}}}^{-}\to {\text{SO}}_{3}^{2-}+{\text{S}}^{\text{2}-},$$

$${\text{S}}_{\text{2}}{{\text{O}}_{\text{3}}}^{\text{2}-}+\text{2H}∙\to \text{S}{{\text{O}}_{\text{3}}}^{\text{2}-}+\text{2}{\text{H}}^{+}+{\text{S}}^{\text{2}-},$$

$${\text{S}}^{\text{2}-}+\text{Z}{\text{n}}^{\text{2}+}+\text{M}{\text{n}}^{\text{2}+}\to \text{ZnS}:\text{M}{\text{n}}^{\text{2}+},$$

$$\text{n}\left(\text{ZnS}:\text{M}{\text{n}}^{\text{2}+}\right)\to {\left(\text{ZnS}:\text{M}{\text{n}}^{\text{2}+}\right)}_{\text{n}}.$$

### Morphology and structure of CS-ZnS and CS-ZnS:Mn^2+ ^QDs

**Figure 2:**
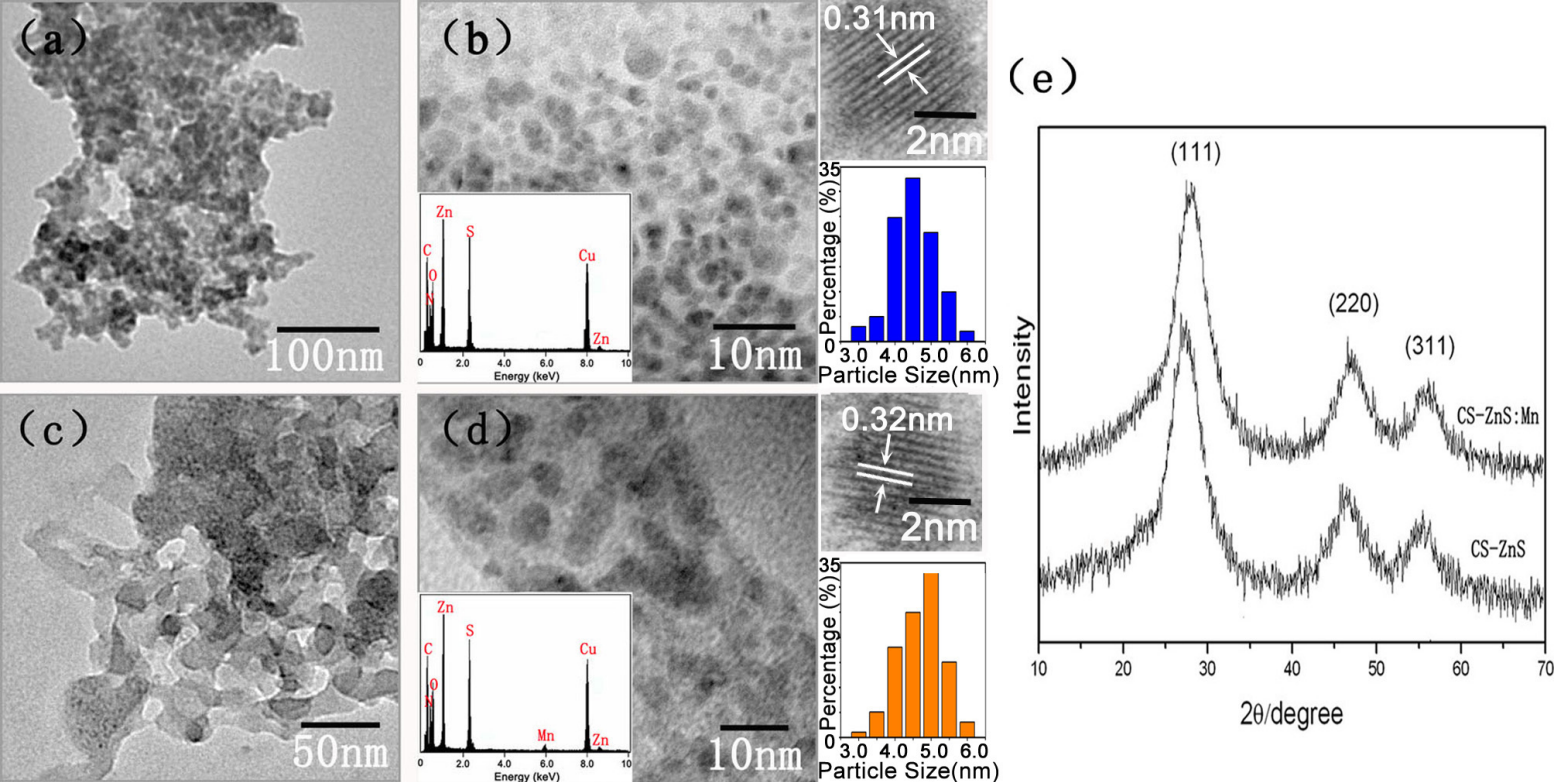
**TEM images, EDS spectra, size distribution, and XRD patterns of QDs fabricated in different conditions**. (**a**) TEM image of ZnS QDs (no chitosan coating); (**b**) TEM image of CS-ZnS (0.1% CS); (**c**) TEM image of CS-ZnS (2% CS); (**d**) TEM image of CS-ZnS:Mn^2+ ^(0.1% CS); (**e**) XRD patterns corresponding to (b) and (d); insets in (b) and (d) are EDS spectra, HRTEM images, and particle size distribution histograms, respectively.

The morphology and structure of CS-ZnS and CS-ZnS:Mn^2+ ^QDs fabricated in different conditions were characterized by TEM, EDS, and XRD. Without chitosan, the ZnS particles were seriously aggregated, and almost no monodispersed ZnS QDs were observed (Figure 2a). When the concentration of chitosan was 0.1% (*w*/*v*), monodispersed CS-ZnS QDs with the mean size of 4.5 nm were observed (Figure 2b). When the concentration of chitosan increased up to 2% (*w*/*v*), the dispersity of QDs was also very poor because of the redundant organic molecules (Figure 2c). The EDS spectrum demonstrated the existence of the Zn, S, C, N, and O elements in CS-ZnS QDs (Figure 2b). The as-synthesized CS-ZnS:Mn^2+ ^QDs were about 5 nm in diameter and consisted of Zn, Mn, S, C, N, and O elements (Figure 2d). The obtained QDs were with narrow particle size distributions (insets in Figure 2b, d). There was no significant difference between the XRD patterns of CS-ZnS and CS-ZnS:Mn^2+ ^QDs (Figure 2e). Three observed peaks could be indexed to (111), (220), and (311) reflections for cubic ZnS (JCPDS no. 77-2100). HRTEM images (insets in Figure 2b, d) indicated that as-synthesized CS-ZnS and CS-ZnS:Mn^2+ ^QDs were highly crystalline, and the distances between the adjacent lattice fringes were measured as about 0.31 nm and 0.32 nm, respectively, which agree well with the literature value for the (111) *d *spacing (JCPDS no. 77-2100). These results indicated that the addition of Mn^2+ ^did not affect the crystal structure of ZnS seriously.

### Fluorescent properties of CS-ZnS and CS-ZnS:Mn^2+ ^QDs

**Figure 3:**
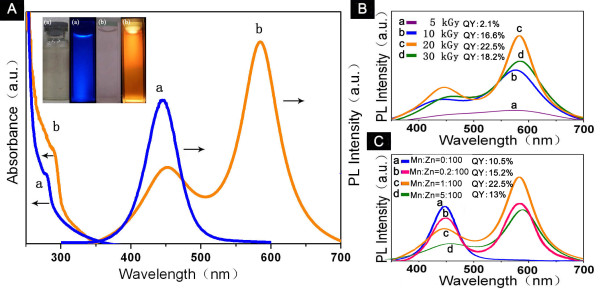
**Fluorescent properties of as-synthesized CS-ZnS and CS-ZnS:Mn^2+ ^QDs**. (**A**) UV-Vis (left) and PL (right) spectra, photographs, (insets) of CS-ZnS QDs (a) and CS-ZnS:Mn^2+ ^QDs (b) prepared in 0.1% CS under 20 kGy (Mn:Zn is 1:100). (**B**) PL spectra and YQs of CS-ZnS:Mn^2+ ^QDs (Mn:Zn is 1:100) prepared in 0.1% CS under different γ-radiation doses, λ_ex _= 290 nm. (**C**) PL spectra and QYs of CS-ZnS:Mn^2+ ^QDs with different doping concentrations prepared in 0.1% CS under 20 kGy, λ_ex _= 290 nm.

As shown in Figure 3A, the absorption peaks of Mn-doped and undoped CS-ZnS QDs were at 295 and 289 nm, respectively. The undoped CS-ZnS QDs showed a PL emission peak at about 447 nm, which is the typical luminescence of ZnS that resulted from the transition of electrons from shallow states near the conduction band to sulfur vacancies present near the valence band. A new strong emission peak at about 590 nm was observed in Mn-doped CS-ZnS QDs. On doping ZnS with Mn^2+^, the Mn^2+ ^substitutes the Zn^2+ ^in the ZnS crystal acting as trap sites, where the electrons and holes can be trapped. An electron can undergo a photoexcitation process in the host ZnS lattice of the nanoparticles and subsequently can decay via a transition from the ^4^T_1 _level to the ^6^A_1 _level, which results in the strong emission at around 590 nm. The photographs of samples (insets of Figure 3A) showed that the as-prepared QDs exhibited excellent water solubility and glowed a bright orange or blue fluorescence under UV illumination, respectively.

Preparation conditions have been proved to be very crucial to the properties of ZnS:Mn^2+ ^[16,17]. Our results indicated that the fluorescence of as-synthesized QDs could be tuned by changing the dose of gamma radiation and the content of Mn in ZnS nanocrystals (Figure 3B, C). The PL emission peak of the sample was not very obvious when the radiation dose was 5 kGy. When the radiation dose was 10 kGy, the QY values of the as-obtained QDs were about 16.6%, and PL emission peaks appeared at around 442 and 584 nm. With the increase of radiation dose, the particle sizes were increasing, and the PL emission peaks appeared a slight red shift. When the radiation dose was 20 kGy, the QY values of the QDs reached 22.5%. However, when the radiation dose increased to 30 kGy, the QY values of the QDs decreased to 18.2% possibly because of the destruction of the crystal structure and chitosan. With the increase of Mn concentrations in ZnS nanocrystals, the PL emission peak around 590 nm was gradually enhanced with a slight red shift, and the QY values of the QDs were also increased. However, when the ratio of Mn to Zn reached 5:100, the QY values of the QDs decreased from 22.5% to 13% again because excessive amounts of Mn^2+ ^in the reaction solution affected the generation and structure of ZnS nanocrystals.

### Cellular labeling and cytotoxicity of CS-ZnS and CS-ZnS:Mn^2+ ^QDs

**Figure 4:**
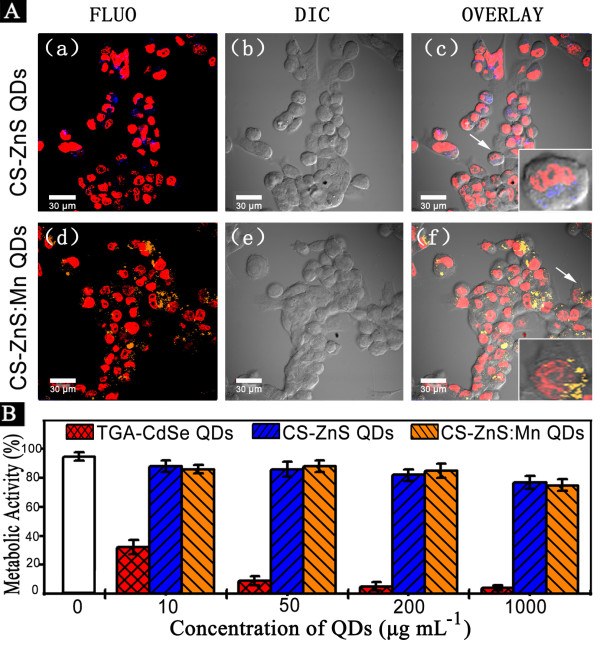
**Cellular labeling and cytotoxicity of CS-ZnS and CS-ZnS:Mn^2+ ^QDs in PANC-1 cells**. (**A**) LSCM images of PANC-1 cells labeled with 10 μg mL^-1 ^of CS-ZnS and CS-ZnS:Mn^2+ ^QDs. Nuclei were stained with 5 μg mL^-1 ^PI. Cells were incubated with QDs for 2 h before imaging. The scale bar represents 30 μm. (**B**) The metabolic activity of PANC-1 cells exposed to TGA-CdSe, CS-ZnS, and CS-ZnS:Mn^2+ ^QDs. The final concentrations of QDs in the medium were 0, 10, 50, 200, and 1,000 μg mL^-1^. Cells were incubated with QDs for 2 h before MTT assays. Data represent mean ± SD for three independent experiments.

LSCM imaging and MTT assays were carried out to examine the cellular labeling and cytotoxicity of as-synthesized QDs. As shown in Figure 4A, CS-ZnS and CS-ZnS:Mn^2+ ^QDs were successfully taken by the PANC-1 cells. Most of the QDs were located in the cytoplasm around the nuclei; only very few QDs were present in the nuclei. Cells containing QDs emitted a bright blue light and orange light, respectively, under laser excitation. The metabolic activity of cells labeled with CS-ZnS and CS-ZnS:Mn^2+ ^QDs were kept above 80% when their concentrations in the medium were below 200 μg mL^-1 ^and were also kept above 70% when their concentrations reached 1,000 μg mL^-1 ^(Figure 4B). The metabolic activity of cells labeled with TGA-CdSe QDs had decreased to 32% when their concentration was 10 μg mL^-1^, which showed a striking contrast. The cytotoxicity of as-prepared QDs was very low, which might be attributed to biocompatible chitosan coatings and nontoxic chemical composition. These results demonstrated the potential application of as-fabricated QDs in biomedical areas.

## Conclusion

In summary, this study has presented an attempt to fabricate biocompatible chitosan-coated ZnS and ZnS:Mn^2+ ^QDs by a γ-radiation technique. The synthesized QDs had good fluorescent properties which could be tuned by changing the dose of gamma radiation and the content of Mn in ZnS nanocrystals. The obtained QDs exhibited low cytotoxicity and high biocompatibility, which might be very useful in further biomedical applications. We claim that this convenient synthesis strategy could be also easily extended to fabricate various other functional nanoparticles coated with chitosan.

## Competing interests

The authors declare that they have no competing interests.

## Authors' contributions

SQC carried out the experimental design and analysis and drafted the manuscript. DC participated in the experimental design. BK and HXZ participated in the synthesis and characterization of quantum dots. YDD participated in the experimental analysis. All authors read and approved the final manuscript.
